# Developing digital biomarker for predicting cognitive response to multi-domain intervention

**DOI:** 10.1038/s41598-026-37123-8

**Published:** 2026-01-30

**Authors:** Ji Hyeun Park, Hyun Sook Kim, Seong Hye Choi, Jee Hyang Jeong, So Young Moon, Yoo Kyoung Park, Chang Hyung Hong, Soo Hyun Cho, Hae Ri Na, Hang-Rai Kim

**Affiliations:** 1 Medical division, Rowan, Cheonan, South Korea; 2 Department of Neurology, CHA Bundang Medical Center, CHA University, Seongnam, South Korea; 3 Department of Neurology, Inha University College of Medicine, Incheon, South Korea; 4 Department of Neurology, Ewha Womans University College of Medicine, Seoul, South Korea; 5 Department of Neurology, Ajou University School of Medicine, Suwon, South Korea; 6 Department of Medical Nutrition (AgeTech-Service Convergence Major), Graduate School of East-West Medical Science, Kyung Hee University, Suwon, South Korea; 7 Department of Psychiatry, Ajou University School of Medicine, Suwon, South Korea; 8 Department of Neurology, Chonnam National University Medical School, Chonnam National University Hospital, Gwangju, South Korea; 9 Department of Neurology, Bobath Memorial Hospital, Seongnam, South Korea

**Keywords:** Digital biomarker, Computerized cognitive training, Mild cognitive impairment, Predictive markers, Cognitive neuroscience

## Abstract

Computerized cognitive training allows real-time tracking of performance metrics that may serve as digital biomarkers. This study investigated the value of a novel in-game digital biomarker, RTACC (Reaction Time-Accuracy Correlation), the correlation between reaction time and accuracy, using data from 130 participants with mild cognitive impairment enrolled in the intervention arm of the SUPERBRAIN-MEET randomized controlled trial. Participants underwent a 24-week multi-domain intervention, consisting of computerized cognitive training, physical exercise, nutritional education, vascular/metabolic risk management, and motivation enhancement. RTACC was derived from task-level RT and accuracy and examined in relation to cognitive and biomarker outcomes. Linear regression analysis revealed a significant association between RTACC and changes in Repeatable Battery for the Assessment of Neuropsychological Status scores from baseline to 24 weeks (beta coefficient = -11.90 ± 3.78, T = − 3.14, *P* = 0.002). RTACC also showed a marginal effect on changes in brain-derived neurotrophic factor levels (beta coefficient = − 3.13 ± 1.64, *P* = 0.057). Logistic regression analysis demonstrated that RTACC combined with clinical information identified good responders with an area under the receiver operating characteristic curve of 0.73 (95% CI: 0.62–0.84). These findings suggest that this in-game digital biomarker (RTACC) may help identify individuals likely to benefit from multi-domain intervention.

**Supplementary Information:**

The online version contains supplementary material available at 10.1038/s41598-026-37123-8.

## Introduction

With the global population aging, the prevalence of cognitive impairment is rising rapidly. Although mild cognitive impairment (MCI) is characterized by measurable cognitive deficits that do not significantly interfere with daily life, MCI is widely regarded as a symptomatic predementia stage, with approximately 10%–15% of individuals with MCI progressing to dementia annually^1,2^.

Research suggests that lifestyle modifications may help reduce the risk of developing dementia in vulnerable populations^3^. The Finnish Geriatric Intervention Study to Prevent Cognitive Impairment and Disability (FINGER) was the first large-scale randomized controlled trial (RCT) to investigate this issue in older adults at increased risk of dementia. The study implemented a multi-domain intervention (MI), including cognitive training, dietary counseling, physical exercise, and vascular and metabolic risk management; over 2 years, the intervention demonstrated positive effects on cognitive function^4^. Similar trials, inspired by the FINGER study, have been conducted in various countries, adapting the intervention to local contexts by considering cultural, dietary, healthcare, and socioeconomic factors^5,6^.

Recently, we conducted the SUPERBRAIN-MEET study (SoUth Korean study to PrEvent cognitive impaiRment and protect BRAIN health through Multi-domain interventions via facE-to-face and vidEo communication plaTforms). This RCT evaluated the effectiveness of MI delivered through both in-person sessions and video communication platforms via a tablet PC application. Findings demonstrated significant cognitive improvement in older adults with MCI^7,8^.

Among various interventions, computerized cognitive training stands out for its game-based tasks designed to enhance a wide range of cognitive functions, including attention, working memory, language and calculation, visuospatial abilities, and executive functions^7,8^. A key advantage of computerized cognitive training over traditional pen-and-paper methods is its ability to monitor in-game data continuously during training sessions. These data can potentially serve as digital biomarkers, enabling early identification of individuals most likely to benefit from cognitive training as well as from broader MI in which cognitive training constitutes a key component.

To date, digital biomarkers from in-game behavioral measures such as reaction time (RT), accuracy, error rates, and learning curves have been used to assess cognitive processing and learning potential^9–11^. However, because these metrics reflect a single behavioral dimension, they are vulnerable to task-specific variability, individual response strategies, and ceiling effects^12,13^. This highlights the need for integrative markers that reliably capture the cognitive processes driving learning and adaptation. The speed–accuracy trade-off (SAT), a fundamental principle of cognitive control describing the inverse relationship between response speed and accuracy provides a theoretical basis for such integrative approaches^14^. Building on this framework, we developed RTACC (Reaction Time–Accuracy Correlation), an integrated behavioral metric that quantifies the task-by-task coordination of speed and accuracy. By applying a joint RT–accuracy metric, RTACC provides a more comprehensive characterization of cognitive efficiency than single-dimension measures, which can be influenced by strategy shifts, practice effects, or ceiling constraints. Notably, prior studies that integrate speed and accuracy components have demonstrated predictive value for cognitive outcomes, further supporting the utility of such multi-dimensional approaches^15^.

In this study, we examined whether RTACC was associated with cognitive outcomes and whether it could help identify individuals who may be more likely to benefit from MI.

## Methods

### Study participants

In this study, data were obtained from participants in the MI group of the SUPERBRAIN-MEET trial (*n* = 130), all of whom were aged 60–85 years, diagnosed with MCI based on Peterson’s criteria^1^ and had at least one modifiable risk factor for dementia, such as hypertension^16^ or diabetes mellitus^17^.

### Study design

This study is a retrospective secondary analysis of data from the SUPERBRAIN-MEET trial (ClinicalTrials.gov Identifier: NCT05023057), a 24-week, multicenter, outcome assessor-blinded RCT with a parallel-group design (MI group and control group) conducted in South Korea from August 2021 to June 2022. MI group participants received a comprehensive 24-week intervention of five key components^7,8^: (i) monitoring and management of metabolic and vascular risk factors, (ii) cognitive training, (iii) physical exercise, (iv) nutritional education, and (v) motivational enhancement.

**Fig. 1:**
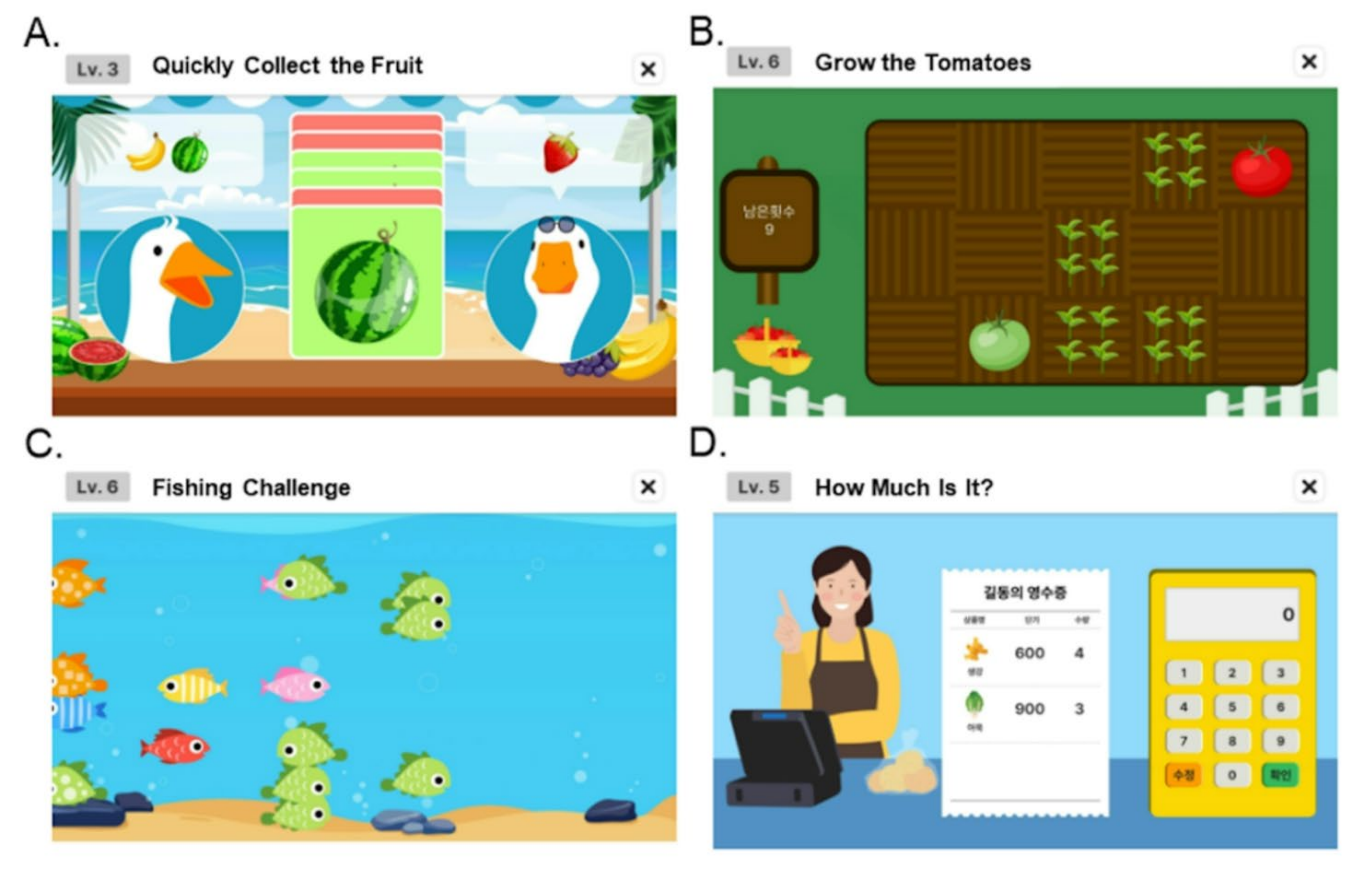
Computerized cognitive training screenshots: (**A**) Quickly Collect the Fruit, (**B**) Grow the Tomatoes, (**C**) Fishing Challenge, (**D**) How Much Is It?.

To facilitate cognitive training, MI group participants were provided a tablet PC equipped with SUPERBRAIN, a computerized cognitive training software designed to enhance attention, working memory, executive functions, visuospatial abilities, and language/calculation. Table 1 provides an overview of the platform’s various cognitive tasks, each specifically designed to target different cognitive functions through engaging, interactive content. Figure 1 displays screenshots of the selected games, highlighting the platform’s user-friendly interface and interactive features.

As a primary outcome, the Repeatable Battery for the Assessment of Neuropsychological Status (RBANS) was measured at baseline, at the 12th week, and at the 24th week, at study completion. Furthermore, as exploratory outcomes, blood biomarkers, such as serum brain-derived neurotrophic factor (BDNF), plasma neurofilament light chain (NFL), plasma glial fibrillary acidic protein (GFAP), and plasma ptau181, were measured at baseline and study completion. Further details on the study participants and design can be found in the original SUPERBRAIN-MEET trial^7,8^.

### Digital biomarker

Throughout the 24-week MI period, participants engaged in computerized cognitive training for approximately 110–160 min per week. Training sessions included a combination of 50-minute in-person group training sessions led by qualified health professionals, 30–40-minute self-administered training at home, and 30–40-minute online training sessions supervised by health professionals. While the specific composition and frequency of sessions varied slightly from week to week, all participants followed a standardized protocol to ensure consistency. Each session comprised four game-based cognitive tasks, each lasting approximately three minutes. Task difficulty was adaptively adjusted based on each participant’s performance in the preceding session to maintain an appropriate level of challenge. Upon completing each cognitive training task, in-game data including RT and accuracy were recorded, generating a continuous dataset for each participant throughout the study period. RT is the interval between stimulus presentation and response initiation, reflecting processing speed^18^. On average, approximately 390 RT–accuracy pairs were recorded per participant. To examine the relationship between these two metrics, we calculated the correlation coefficient between RT and accuracy (RTACC) as follows: RTACC=corr(RT_i_, Accuracy_i_)

where RT_i_ denotes the mean RT for task i (3-minute duration), and Accuracy_i_ denotes the proportion of correct trials out of the total trials during the 3-minute task i.

All data from the computerized cognitive training tasks underwent preprocessing before analysis. We first assessed the dataset for missing values, and found no missing RT or accuracy data. Outliers were then identified using a robust MAD-based rule^19^, but no data met this criterion and therefore no outliers were removed.

We then investigated whether RTACC could serve as a digital biomarker to predict cognitive outcomes and identify individuals responding favorably to MI.

### Statistical analyses

#### Association with the change of RBANS

To assess the relationship between an individual’s RTACC of 24-week continuous dataset and changes in RBANS from baseline to 24 weeks, we conducted linear regression analysis, adjusting for baseline RBANS, age, sex, years of education, and *APOE* ɛ4 genotype as covariates: RBANS (at 24 weeks) = β0 + β1 age + β2 sex + β3 years of education + β4 *APOE* ɛ4 + β5 RBANS (baseline) + β6 RTACC.

### Sensitivity analyses

To assess whether the length of data used influences the association between RTACC and changes in RBANS, we performed a sensitivity analysis, calculating RTACC over different time periods: initial 1 week, 2 weeks, 3 weeks, 6 weeks, and 12 weeks. Next, to determine whether specific cognitive training content influences the association between RTACC and changes in RBANS, we conducted another sensitivity analysis, systematically excluding each type of training content and assessing the impact of exclusion on the association. Finally, in order to reduce the influence of potential outliers, we performed robust linear models (RLM).

### Pathway analysis using blood biomarkers

Next, in order to evaluate mediating factors between RTACC and cognitive outcome, we performed structural equation modeling (SEM). We first built a model in which RTACC had direct and indirect paths to cognitive outcome through all possible blood biomarkers. After path coefficients were derived, the paths were examined with P-values to achieve a more parsimonious model.

### Identification of good responders to MI

To evaluate whether RTACC can predict a good responder to MI, we conducted logistic regression analysis. We defined a good responder as, after 24 weeks, having an increased RBANS score and a poor responder as having a decreased RBANS score. We performed a receiver operating characteristic curve analysis and measured the area under the receiver operating characteristic curve (AUC).

We defined the P-value < 0.05 as statistically significant. MATLAB (MathWorks R2020a, Natick, MA, USA) and SPSS 21 (SPSS Inc., Chicago, IL, USA) were used for statistical analyses and visualization of results.

### Ethical approval and consent

SUPERBRAIN-MEET trial (NCT05023057) was conducted in accordance with the International Conference on Harmonization Good Clinical Practice Guidelines. The study protocol was approved by the institutional review boards of all participating hospitals: Ajou University Hospital (AJIRB-BMR-SUR-21–323), Ewha Womans University Seoul Hospital (SEUMC-2021-07-037), Ewha Womans University Mokdong Hospital (EUMC-2021-08-003), Bobath Memorial Hospital (P01-202109-11-002), Chonnam National University Hospital (CNUH-2021-326), CHA Bundang Medical Center (CHAMC-2022-01-020), Catholic Kwandong University International St. Mary’s Hospital (IS22EIMI0008), Konkuk University Hospital (KUMC-2021-08-023), Uijeongbu St. Mary’s Hospital (UIRB-2022-0428), Pusan National University Hospital (2108-016-015), Jeonbuk National University Hospital (CUH-2021-08-043), Hanyang University Hospital (HYUH-2021-07-041), Inha University Hospital (INHAUH-2021-06-040), Dong-A University Hospital (DAUHIRB-21–168), Myongji Hospital (MJH-2021-08-032), Samsung Medical Center (SMC-2021-08-022), and Eulji University Hospital (EMC-2021-12-004). The written informed consent was obtained from all participants prior to enrollment.

### Data availability

The data used in this study were obtained from the SUPERBRAIN-MEET trial. Due to privacy and ethical restrictions, the datasets are not publicly available. Access to the dataset is available from the corresponding author of SUPEPBRAIN-MEET trial on reasonable request.

## Results

The intervention group consisted of 130 participants, whose demographic characteristics are displayed in Table 2. The mean change in RBANS scores from baseline to the 24th week was 8.26 (± 8.74).

### Association with the change of RBANS

**Fig. 2:**
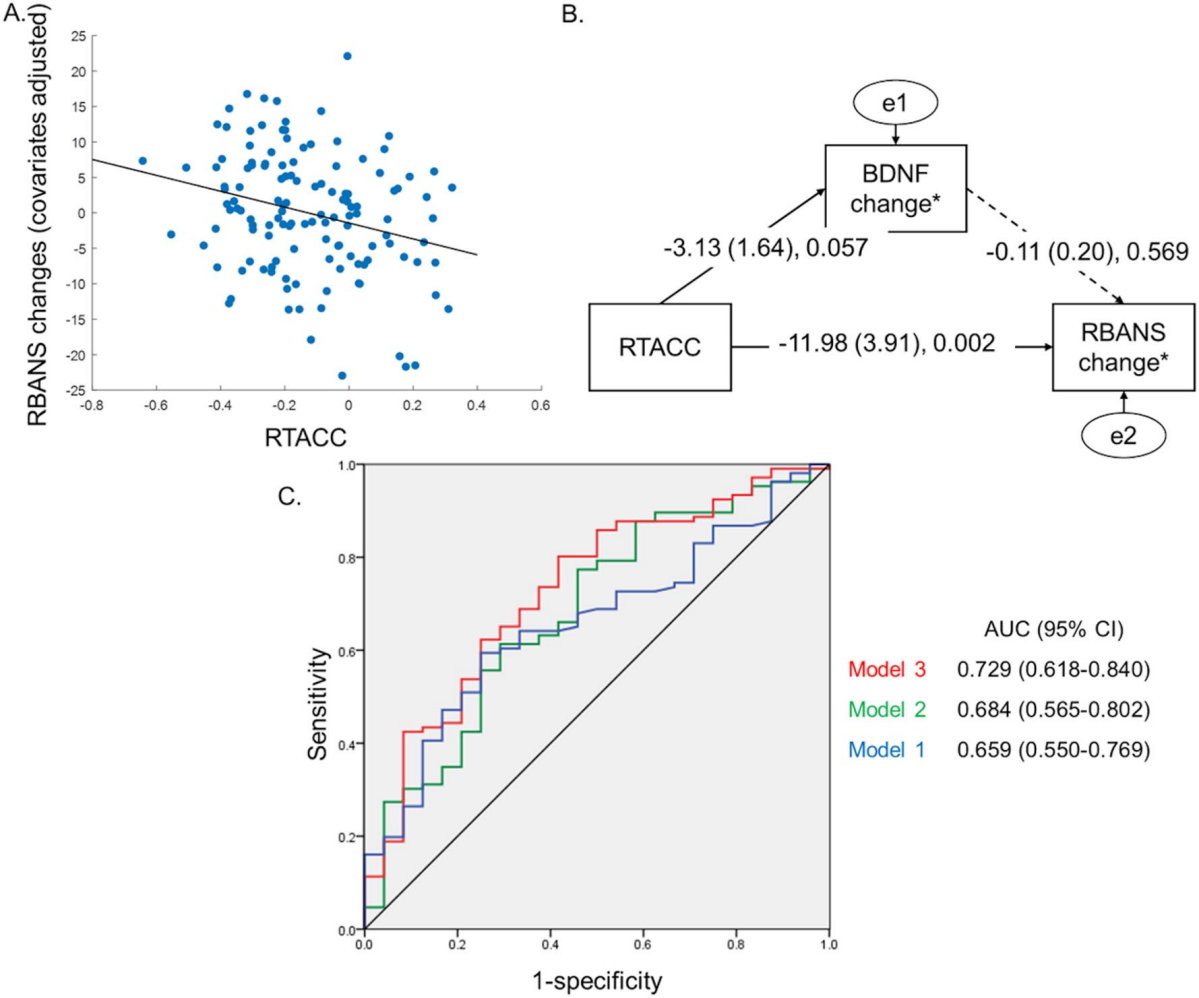
(**A**) Scatter plot of RBANS changes and RTACC, (**B**) Path analysis of RBANS changes, RTACC, and blood biomarker, and (**C**) ROC curve for prediction of good responders. Model 1 = clinical information (age, sex, education, and *APOE* ɛ4 genotype), Model 2 = RTACC, Model 3 = clinical information + RTACC BDNF, Brain-Derived Neurotrophic Factor; RBANS, Repeatable Battery for the Assessment of Neuropsychological Status; RTACC, correlation coefficient of reaction time and accuracy.

In the linear regression model, RTACC was significantly associated with the change in RBANS scores (beta coefficient =−11.90 (± 3.78), t-value = − 3.14, P-value = 0.002). According to the estimated beta coefficient for RTACC, a negative RTACC value was associated with a greater increase in RBANS scores in the 24th week (Table 3; Fig. 2A).

### Sensitivity analyses

Results from the first sensitivity analysis assessing how the data’s length influenced the association between RTACC and changes in RBANS showed that this association became evident after 2 weeks (Table 3). Findings from the second sensitivity analysis examining specific cognitive training’s impact revealed that the association between RTACC and changes in RBANS remained significant, regardless of which training content was excluded (Table 4). Finally, a sensitivity analysis using RLM showed similar results that there were a significant association between RTACC and changes in RBANS (supplementary Tables 1 and 2).

### Pathway analysis using blood biomarkers

In the SEM analysis, the final model indicated that decreased RTACC values (i.e., more negative values) were associated with increased RBANS change (beta coefficient = − 11.98 ± 3.91, P-value = 0.002). Additionally, decreased RTACC was marginally associated with increased serum BDNF change (beta coefficient = − 3.132 ± 1.64, P-value = 0.057). However, the pathway from the BDNF change as well as pTau, NFL and GFAP to the RBANS change was not significant (beta coefficient = − 0.118 ± 0.20, P-value = 0.569) (Fig. 2B; Table 5).

### Identification of good responders to MI

We developed prediction models to evaluate RTACC’s clinical utility for identifying good responders to MI. In our dataset, 106 participants showed an increased RBANS score after MI (good responders). Model 1, which included only clinical information (age, sex, education, and *APOE* ɛ4 genotype), showed an AUC of 0.65 (95% confidence interval [CI] 0.55–0.77). Model 2, which included the RTACC value, showed an AUC of 0.68 (95% CI 0.56–0.80). Finally, model 3, which included both clinical information and RTACC, showed an AUC of 0.73 (95% CI 0.62–0.84) (Fig. 2C).

## Discussion

In the present study, we developed a novel digital biomarker derived from in-game performance, which was associated with cognitive outcomes. Our main findings are as follows: First, RTACC values were significantly associated with changes in RBANS scores; this relationship remained robust across varying lengths of data collection and regardless of cognitive training content. Second, RTACC values showed a marginal association with changes in BDNF levels. Finally, RTACC combined with clinical information showed predictive capability for identifying good responders to MI.

Rather than relying on single-dimension digital biomarkers, we applied a joint RT–accuracy metric to derive a more comprehensive index of processing efficiency. Digital biomarkers have also been derived from user engagement indices such as training frequency, cumulative training time, and adherence, which primarily quantify training dose^20,21^. Physiological signals from electroencephalography (EEG) or eye tracking similarly provide markers of neural or attentional states relevant to training response^22,23^. Unlike engagement metrics that index training quantity, RTACC captures the quality of task performance, and unlike EEG- or eye tracking–based biomarkers, it requires no external hardware, enabling scalable implementation in real-world and home-based intervention environments.

Notably, our findings indicate that participants with negative RTACC values were more likely to show greater improvements in RBANS scores following MI. Typically, faster RT in cognitive tasks is associated with reduced accuracy, and older adults, in particular, are known to slow their responses intentionally to minimize errors^24,25^. However, our results revealed a distinct pattern: participants who demonstrated both faster RT and higher accuracy during training showed greater improvements in RBANS scores.

Such patterns, in which speed and accuracy improve concurrently (a negative correlation between RT and accuracy), have been described in prior work. Within the drift-diffusion model (DDM), this corresponds to higher drift rates, indicating more efficient evidence accumulation and more effective task engagement^26–28^. This improvement likely reflects increased task automatization, enabling individuals to process information more efficiently and overcome the constraints of the traditional SAT^29^. Accordingly, the association between RTACC and RBANS improvement observed in this study likely reflects true cognitive enhancement^30^.

Sensitivity analyses revealed that RTACC values obtained during the initial 2 weeks of training were associated with subsequent changes in RBANS scores. This finding suggests that early RTACC values may serve as valuable monitoring biomarkers^31^ for tailoring interventions. By enabling early identification of individuals at risk of poor cognitive outcomes, this approach allows timely modifications to improve intervention outcome. Specifically, individuals predicted to have poor outcomes based on early RTACC values could benefit from adjustments to the intervention program or the incorporation of additional support strategies, while those predicted to show favorable outcomes should be encouraged to maintain their current regimen. Nevertheless, the validity of this personalized approach based on RTACC should be tested in future RCT.

Notably, the association between the RTACC and RBANS scores remained robust regardless of the training contents that were included. This indicates that the observed relationship is not attributable to a specific training component but rather reflects the multiple training contents’ cumulative effect.

In the pathway analysis, RTACC demonstrated a statistically significant direct effect on changes in RBANS scores and a marginally significant direct effect on changes in BDNF levels (*P* = 0.057). BDNF is a central regulator of neuroplasticity, promoting synaptic transmission, dendritic growth, and long-term potentiation, processes that are fundamental to learning and memory^32^. Multiple studies have shown that cognitive and physical interventions increase BDNF expression alongside improvements in cognitive performance, including enhanced accuracy and RT^33,34^. Because RTACC is a digital biomarker derived from accuracy and RT, changes in RTACC may reflect underlying BDNF-mediated neuroplastic mechanisms. However, the association between RTACC and changes in BDNF levels was only marginally significant and is therefore treated as exploratory. These biological findings should be replicated in larger samples before any firm conclusions are drawn about mechanistic pathways.

We demonstrated that the digital biomarker RTACC, when combined with clinical information, could identify individuals likely to respond well or poorly to the intervention, achieving an AUC of 0.73 (95% CI 0.62–0.84). Previous studies have focused primarily on predicting intervention outcomes based on baseline clinical information such as sociodemographic factors and genetic markers^35,36^. However, these factors are not dynamic and serve as indirect indicators. In contrast, RTACC is a real-time marker generated during the training process, directly reflecting the individual’s learning performance.

This study has several limitations. First, the sample size was small, and the results were not validated in an independent dataset. Because the prediction model was evaluated without internal cross-validation or external testing, the reported classification performance may be optimistic and the possibility of overfitting cannot be excluded. External validation in larger and more heterogeneous populations is required to establish the robustness and generalizability of RTACC as a digital biomarker. Second, because RTACC was evaluated within MI, the integrated design limits isolation of the independent effects of cognitive training. Therefore, the observed cognitive changes should be interpreted as reflecting the overall impact of the MI rather than any single component.

In conclusion, this study developed a novel digital biomarker (RTACC) based on in-game performance, which was associated with cognitive outcomes. This tool holds promise for identifying individuals who respond most effectively to MI, opening up the possibility of providing personalized treatment approaches for each patient.

**Table 1:** Computerized cognitive training in SUPERBRAIN and associated targeted cognitive functions.

Domain	Game name	Task description
Attention	Tap the Circles in Order	Memorize circle order and tap accordingly.
	Tap the Numbers in Order	Tap numbers in ascending order.
	Press the Number in Reverse Order	Tap numbers in descending order.
Working memory	Tap the Circles in Reverse Order	Memorize circle order and tap in reverse.
	Grow the Tomatoes	Memorize and recall crop positions.
	Pair Matching	Match hidden cards by remembering pictures.
Executive function	Quickly Collect the Fruit	Identify and click matching fruit card.
	Remember the Previous Card	Compare cards based on given rule.
Visuospatial ability	Treasure Hunt	Follow arrows to locate hidden treasures.
	Spot the Difference	Identify the flipped image among rotated ones.
	Fishing Challenge	Catch correct fish based on visual cues.
	Merge the Shapes	Predict final shape when pieces are combined.
	Colorful Box Sorting	Match color sequences quickly and accurately.
	Touch-Touch Card Game	Tap cards matching multiple conditions.
Language /Calculation	When Will It Arrive?	Read train tickets and calculate arrival times.
	How Much Is It?	Add receipt items and input total.
	Reverse Calculation	Mentally rotate and solve inverted math.
	Crack the Honeycomb	Complete number grid under constraints.

**Table 2:** Demographic characteristics of 130 participants at baseline.

Demographics	Mean (SD or %)
Years of age	73.02 (± 5.53)
Education years	10.73 (± 4.54)
Female, n	83 (63.85)
*APOE* ɛ4, n	41 (31.54)
RBANS baseline	90.10 (± 15.26)
RBANS at 24th week	98.37 (± 16.51)
RBANS changes	8.26 (± 8.74)

APOE, apolipoprotein E; RBANS, Repeatable Battery for the Assessment of Neuropsychological Status.

**Table 3:** Association of RTACC and change of RBANS over difference time period (Sensitivity analyses).

Time period of RTACC	β coefficient (SE)	t-statics	*P*-value
24 weeks	−11.90 (± 3.78)	−3.14	0.002
< 12 weeks	−9.50 (± 2.96)	−3.21	0.001
< 6 weeks	−7.67 (± 2.64)	−2.89	0.004
< 3 weeks	−5.29 (± 2.24)	−2.36	0.019
(< 2 weeks	−5.79 (± 2.01)	−2.87	0.004
< 1 week	−2.85 (± 1.66)	−1.71	0.08

RBANS, Repeatable Battery for the Assessment of Neuropsychological Status; SE, standard error.

**Table 4:** Association of RTACC after excluding each type of game content (Sensitivity analyses).

Domain	Excluded game name	β coefficient (SE)	t-statics	*P*-value
Attention	Tap the Circles in Order	−11.87 (± 4.08)	−2.90	0.004
	Tap the Numbers in Order	−11.8 (± 3.75)	−3.14	0.002
	Press the Number in Reverse Order	−11.64 (± 3.71)	−3.13	0.002
Working memory	Tap the Circles in Reverse Order	−11.64 (± 3.92)	−2.97	0.003
	Grow the Tomatoes	−11.13 (± 3.54)	−3.14	0.002
	Pair Matching	−12.58 (± 3.93)	−3.19	0.001
Executive function	Quickly Collect the Fruit	−10.79 (± 3.65)	−2.95	0.003
	Remember the Previous Card	−11.39 (± 3.67)	−3.09	0.002
Visuospatial ability	Treasure Hunt	−10.5 (± 3.71)	−2.82	0.005
	Spot the Difference	−10.89 (± 3.65)	−2.98	0.003
	Fishing Challenge	−11.33 (± 3.7)	−3.06	0.002
	Merge the Shapes	−11.96 (± 3.73)	−3.20	0.001
	Colorful Box Sorting	−12.08 (± 3.68)	−3.28	0.001
	Touch-Touch Card Game	−12.03 (± 3.71)	−3.24	0.001
Language /Calculation	When Will It Arrive?	−10.78 (± 3.83)	−2.81	0.005
	How Much Is It?	−11.8 (± 3.73)	−3.16	0.001
	Reverse Calculation	−11.75 (± 3.75)	−3.13	0.002
	Crack the Honeycomb	−11.9 (± 3.7)	−3.21	0.001

SE, standard error.

**Table 5:** Results of SEM between RBANS change, RTACC, and blood biomarker.

Blood biomarker	RTACC◊Blood biomarker β coefficient (SE), *P*-value	Blood biomarker◊RBANS change β coefficient (SE), *P*-value
pTau	2.832 (± 4.22), 0.503	−0.014 (± 0.081), 0.863
BDNF	−3.132 (± 1.64), 0.057	−0.118 (± 0.20), 0.569
NfL	3.879 (± 7.325), 0.596	0.12 (± 0.47), 0.793
GFAP	−31.69 (± 25.75), 0.218	−0.015 (± 0.013), 0.271

BDNF, Brain-Derived Neurotrophic Factor; GFAP, Glial Fibrillary Acidic Protein; NfL, neurofilament light chain; pTau, phosphorylated Tau, SE, standard error.

## Supplementary Information

Below is the link to the electronic supplementary material.

Supplementary Material 1

## Data Availability

The data used in this study were obtained from the SUPERBRAIN-MEET trial. Due to privacy and ethical restrictions, the datasets are not publicly available. Access to the dataset is available from the corresponding author of SUPEPBRAIN-MEET tiral on reasonable request.
