# Supplementary material for: Developing digital biomarker for predicting cognitive response to multi-domain intervention
Source: Sci Rep. 2026 Jan 30;16:6730. doi: 10.1038/s41598-026-37123-8 (PMC12913615; doi:10.1038/s41598-026-37123-8)
Supplement: Supplementary file 1 — Supplementary Material 1 [file 41598_2026_37123_MOESM1_ESM.docx]

**Supplementary Table**

**Supplementary Table 1. Robustfit model results for the association of RTACC and change of RBANS over difference time period**

| **Time period of RTACC** | **β coefficient (SE)** | **t-statics** | **P-value** |
| --- | --- | --- | --- |
| 24 weeks | -11.29 (±3.95) | -2.85 | 0.0050 |
| <12 weeks | -8.97 (±3.09) | -2.90 | 0.0044 |
| <6 weeks | -7.61 (±2.77) | -2.74 | 0.0069 |
| <3 weeks | -5.30 (±2.30) | -2.29 | 0.0231 |
| (<2 weeks | -6.06 (±2.07) | -2.92 | 0.0041 |
| <1 week | -2.60 (±1.72) | -1.51 | 0.1329 |

RBANS, Repeatable Battery for the Assessment of Neuropsychological Status; SE, standard error

**Supplementary Table 2. Robustfit mode results for the association of RTACC after excluding each type of game content**

| **Domain** | **Excluded game name** | **β coefficient (SE)** | **t-statics** | **P-value** |
| --- | --- | --- | --- | --- |
| Attention | Tap the Circles in Order | -11.1527 (±3.9211) | -2.84428 | 0.0052 |
|  | Tap the Numbers in Order | -10.9608 (±3.8775) | -2.82679 | 0.0055 |
|  | Press the Number in Reverse Order | -11.3168 (±4.2525) | -2.66121 | 0.0088 |
| Working memory | Tap the Circles in Reverse Order | -10.9693 (±4.1099) | -2.669 | 0.0086 |
|  | Grow the Tomatoes | -10.5777 (±3.7027) | -2.85675 | 0.005 |
|  | Pair Matching | -12.6859 (±4.0831) | -3.10692 | 0.0023 |
| Executive function | Quickly Collect the Fruit | -10.0645 (±3.8391) | -2.62159 | 0.0099 |
|  | Remember the Previous Card | -10.7888 (±3.8389) | -2.81041 | 0.0058 |
| Visuospatial ability | Treasure Hunt | -9.9713 (±3.8629) | -2.58132 | 0.011 |
|  | Spot the Difference | -10.0037 (±3.8458) | -2.60119 | 0.0104 |
|  | Fishing Challenge | -10.7784 (±3.8683) | -2.78633 | 0.0062 |
|  | Merge the Shapes | -11.2381 (±3.904) | -2.87863 | 0.0047 |
|  | Colorful Box Sorting | -11.519 (±3.8436) | -2.99696 | 0.0033 |
|  | Touch-Touch Card Game | -11.3931 (±3.8949) | -2.92514 | 0.0041 |
| Language /Calculation | When Will It Arrive? | -9.9816 (±4.0039) | -2.49296 | 0.014 |
|  | How Much Is It? | -11.1598 (±3.9045) | -2.85818 | 0.005 |
|  | Reverse Calculation | -11.0186 (±3.9248) | -2.80746 | 0.0058 |
|  | Crack the Honeycomb | -11.2266 (±3.8805) | -2.89308 | 0.0045 |

SE, standard error
